# Impact of elbow stiffness on running economy in trained athletes

**DOI:** 10.1177/17585732241306369

**Published:** 2024-12-19

**Authors:** Cecilia Wilk, Cyril Besson, Laurie Stockton, Frédéric Vauclair, Vincent Gremeaux

**Affiliations:** 1 University of Lausanne, Lausanne, Switzerland; 2 Sports and Exercise Medicine Center, Swiss Olympic Medical Center, Lausanne University Hospital, Lausanne, Switzerland; 3 Lausanne University Sports Sciences Institute, University of Lausanne, Lausanne, Switzerland; 4 Orthopedic Surgery and Traumatology, Lausanne University Hospital, Lausanne, Switzerland; 5 Bone and Motion Center, Hirslanden Clinique Bois-Cerf, Lausanne, Switzerland

**Keywords:** elbow movement, running performance, biomechanics, endurance, joint stiffness, running efficiency

## Abstract

**Background:**

Elbow injuries are likely to generate a decreased range of motion (ROM), which might negatively affect athletic performance. To date, the effect of elbow stiffness on endurance running performance has never been studied. We conducted an observational, prospective, cross-over study to examine the impact of elbow stiffness on running economy.

**Methods:**

Twenty trained athletes performed running economy tests at 12 km·h^−1^, with and without a limited elbow ROM (flexion: 90°, extension: 45°), imposed by a dynamic brace mimicking a severe elbow stiffness. Relative intensity and performance indexes were measured during a subsequent maximal incremental exercise test.

**Results:**

Running economy was measured at 180 ± 10.6 mlO_2_·km^−1^·kg^−1^ with a full ROM, and 180.2 ± 12.3 mlO_2_·km^−1^·kg^−1^ with the limited ROM showing a non-significant 0.1% difference (*p* = 0.871).

**Discussion:**

Athletes experiencing post-traumatic elbow stiffness can find reassurance in knowing that it does not seem to impact a crucial metric of endurance running performance, namely running economy. Further research could explore elbow movement at different intensities of running, from higher aerobic speeds to sprinting.

## Introduction

Due to its unique anatomical and mechanical structure, the elbow joint is prone to stiffness after injury or surgery.^1^ Actually, 5% of elbow trauma result in stiffness, regardless of the type of treatment used.^2^ Elbow stiffness is defined by a decreased range of motion (ROM), with an extension deficit greater than 30° and a flexion less than 120°.^3,4^ This condition has a great impact on the patient's quality of life by limiting some of the movements commonly used for everyday or professional activities, such as dressing, cooking or any manual work.

When considering sports activities, reduced elbow ROM can also alter participation. The impact of this deficiency has mainly been investigated in throwing or racket sports. Indeed, a reduced elbow ROM significantly impacts the throwing technique in baseball players.^5^ However, sports that do not involve as much upper limb movement, such as running, might also be influenced by elbow stiffness. Tartaruga et al. investigated the biomechanics of running and found a positive association between the elbow's ROM and running economy (RE).^6^ RE measures were performed on 16 participants running for 6 min at 16 km·h^−^1 and was correlated with biomechanical variables like vertical oscillation of the centre of mass or stride length, balance time and elbow ROM. Their mean elbow ROM was determined in a kinematic analysis using reflective markers on the shoulder, elbow and wrist, and was found to be of 38.8 ± 12.6°.^6^

RE is one of the three determining factors of performance in endurance running, together with maximal oxygen consumption ( $\dot{V}$ O_2_max) and the fraction of maximum oxygen uptake that can be sustained for a prolonged period.^7,8^ RE is defined as the steady-state oxygen consumption at a specific running speed, offering insight into the energy expenditure of the athlete while running at a submaximal pace. An athlete with better RE will use less oxygen to run at the same speed than another athlete who has poor RE.^9^ Few studies have investigated the importance of upper limb kinematics on RE. These studies demonstrated that restricting arm swing by placing the hands-on top of the head detrimentally impacts RE, whereas findings were inconclusive with other methodologies.^10–12^

Numerous papers investigated the impact of global arm swing on running, but none of them focused solely on elbow movement. In fact, most of these studies looked at the impact of shoulder stiffness on running performance.^9,11–15^ To the best of our knowledge, no study has explored the impact of elbow stiffness on running performance. This study aimed to investigate the impact of elbow stiffness on RE, hypothesizing that it would adversely affect this critical parameter of running performance.

## Materials and methods

### Design

We conducted an observational, prospective, cross-over study involving participants who performed running trials with and without restricted elbow ROM. All measurements were obtained within a single session, during which participants completed two submaximal tests—one with and one without ROM restriction—alongside a maximal incremental test.

### Participants

Twenty participants were included in the study, after giving their informed written consent. Inclusion criteria were:  ≥ 18 years old, male, healthy,  ≥ 2 years of running experience,  ≥ 3 trainings per week and ability to run 10 km in less than 50 min. These athletes are all part of the tier 2 in the up-to-date participation classification framework reference, defined by a regular training ∼3× per week, participation in regional competitions, identifying with a specific sport and training for competition purposes.^16^ Exclusion criteria were: having suffered from an injury or surgery on the elbow, suffering from a reduced ROM of the upper limb, impaired capacity for discernment, being sick or injured at the time of the evaluation and any cardiorespiratory or orthopaedic condition limiting running ability.

The ethics committee of the Canton de Vaud approved the study (#2021-01830). All procedures were conducted according to the Helsinki Declaration.

### Methodology

**Figure 1.**
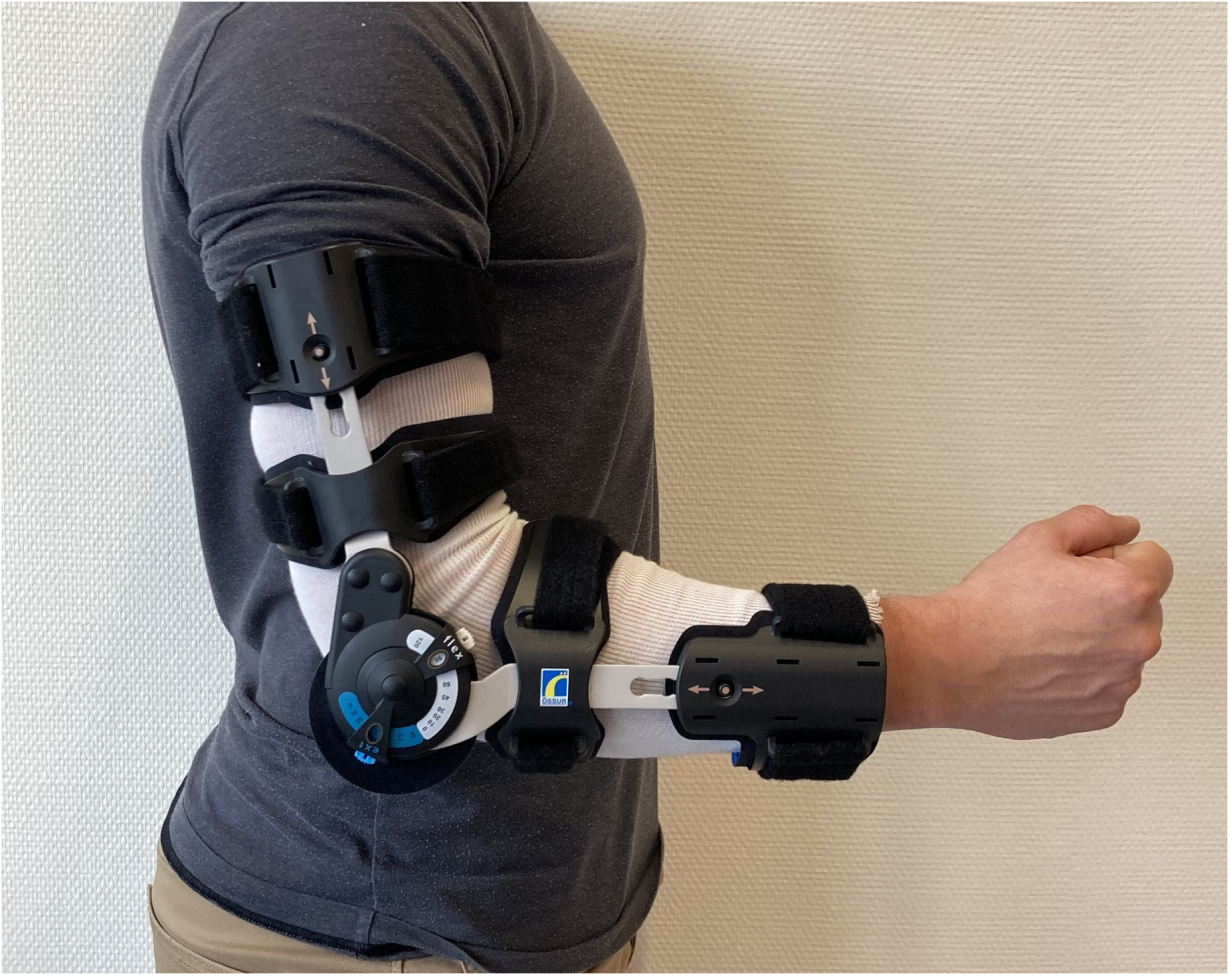
Set-up illustration.

Participants came to the physiology laboratory of the sports medicine unit of Lausanne University Hospital under standardized conditions (no training or strenuous physical activity 48 h before the visit, no snacks 1 h before the visit, limitation of alcohol and coffee consumption in the last 24 h). Height and weight were measured, and participants were equipped with a heart rate monitor (Polar H10, Polar Electro Oy, Finland) and a silicone mask with sensors connected to a metabolic cart (Metalyzer 3b, Cortex Biophysik GmbH, Germany) allowing analysis of cardiorespiratory responses (oxygen consumption (V˙O_2_), carbon dioxide production (V˙CO_2_), ventilation (V˙E) and heart rate (HR)). The reliability of the used metabolic cart (Metalyser 3b) has been evaluated, showing an accuracy of  ± 3% in all volume measures, which is deemed reliable.^17,18^ The set-up is illustrated in Figure 1.

After a standing rest period of 3 min on the treadmill (HP Cosmos, Pulsar, Germany), each participant warmed up for 5 min at a running speed of 10 km·h^−1^. Two 5-min steady-state RE measurements at 12 km·h^−1^ were then performed with a 3-min break in between. One of the measurements was done with a free elbow ROM and the other one with a restricted ROM (flexion: 90°, extension: 45°) as to replicate a severe elbow stiffness,^1^ with an elbow hinged brace (Össur, Reykjavík, Island) placed on the right arm. The dominant hand was not determined prior to testing, all participants wore the brace on the right arm. The order of tests was previously randomly determined. At the beginning and end of each test, a capillary blood sample was taken from the fingertip to measure the blood lactate concentration ([La]) using a Biosen C-Line analyzer (EKF Diagnostics, Cardiff, Wales). The RE was calculated using the average V˙O_2_ of the last 2 min of the test divided by the running speed, to obtain a value in ml·km^−1^·kg^−1^.

After a 5-min break, participants performed a maximal incremental running test without elbow movement limitation. The protocol began at 9 km·h^−1^ and the speed increased by 0.5 km·h^−1^ each minute until maximal voluntary exhaustion was reached. Instructors provided encouragement to ensure that maximum effort was achieved. Participants had to meet three out of five of the following criteria for their test to be considered as maximal: voluntary exhaustion, peak HR within 10 beats·min^−1^ of the maximum expected for age, peak respiratory exchange ratio  > 1.10, plateau of the V˙O_2_-velocity relationship with V˙O_2_ increasing by <2 ml·min^−1^·kg^−1^ despite increasing velocity and maximal lactate above 8 mmol·l^−1^. The peak incremental test speed reached at the end of the test was designated as V_incrtest_. The velocity values at which the V˙O_2_ plateau began were retained as the maximum aerobic speed. Gas exchange variables were averaged over 20 s. The highest V˙O_2_ was retained as the V˙O_2peak_.

The peak incremental test aimed to describe the population performance, as well as the relative intensity of 12 km·h^−1^ speed chosen for the RE test. Lactate levels were used to make sure that the RE test speed remained submaximal (<VT2) for reliable evaluation of RE.

The ventilatory thresholds (VTs) were calculated with the data collected during the peak incremental test by two investigators performing a consensual analysis. The first ventilatory threshold (VT1) was determined with the following criteria: (1) excess V˙CO_2_ relative to V˙O_2_ above the VT1 with the modified V-slope method, (2) identifying hyperventilation relative to oxygen, (3) excluding hyperventilation relative to CO_2_ at the VT1 inflection point identified by criteria 1 and 2.^19^ The second ventilatory threshold (VT2) was determined with the following criteria: (1) increase in both respiratory equivalent (V˙E/V˙O_2_ and V˙E/V˙CO_2_), (2) a decrease in PETCO_2_, (3) a loss of linearity from V˙E/V˙CO_2_ plots.^20,21^

### Statistical analysis

#### Sample size calculation

We deemed the difference between two RE measures to be clinically significant at 3%.^22^ Given an alpha risk of 0.05 (5%) and a beta risk of 10%, we calculated that 18 participants were required to test the hypothesis that RE with limited elbow ROM differs significantly from that with unrestricted elbow ROM. The calculation was based on an estimated average RE of our subjects at 190 mlO_2_·km^−1^·kg^−1^.^23^ To accommodate potential exclusions or drop-outs, we opted to include 20 participants.

#### Statistical analysis plan

The Kolmogorov–Smirnov test was used to test the normality of the data collected. Data are presented as mean ± SD. For comparisons a paired *t*-test was performed as all data passed normality test. All the statistical analyses were performed with GraphPad Prism (Boston, USA, V8.3.0).

## Results

### Participants’ performance level

The participants comprised mostly experienced runners, with their primary disciplines being trail running or road running (17 subjects), cycling (2), and hockey (1). Maximal incremental test was performed to assess that all participants were healthy recreational athletes with a relative peak VO_2_ of 48.1 ± 6.2 mlO_2_·min^−1^·kg^−1^. All participants produced maximal effort. Characteristics and maximal performance test results are presented in Table 1.

**Table 1.** Participant's characteristics.

Age [y]	40 ± 8
Body mass [kg]	73.1 ± 8.3
Stature [cm]	176.2 ± 6.1
BMI [kg·cm^−2^ ]	23.5 ± 2.1
ROM right elbow [°] (F/E)	141 ± 3 / −2 ± 4
Training [h per week]	6.4 ± 4.5
Training [km per week]	31.9 ± 15.0
Training [sessions per week]	3.6 ± 1.4
Peak $\dot{V}$ O_2_ [l·min^−1^]	3.55 ± 0.43
Peak $\dot{V}$ O_2_ [mlO_2_·min^−1^·kg^−1^]	48.1 ± 6.2
HR peak [b·min^−1^]	185 ± 14
$\dot{V}$ E peak [l·min^−1^]	143.3 ± 17.5
V_incrtest_ [km·h^−1^]	16.9 ± 1.3
MAS [km·h^−1^]	16.6 ± 1.3

BMI: body mass index; ROM: range of motion; Peak $\dot{V}$ O_2_: maximal oxygen consumption; HR peak: heart rate at maximal effort; $\dot{V}$ E peak: minute ventilation at maximal effort; V_incrtest_: the final (peak) incremental test speed reached at the end of the test; MAS: maximal aerobic speed.

The RE test was actually in a submaximal state for all participants, with 12 km·h^−1^ being a speed settled in between the two VTs (see Table 2), and lactate level remaining below 4 mmol·l^−1^.^24,25^ Relative intensity was closer to VT1 than VT2, as the percentage of peak $\dot{V}$ O_2_ which was 74.3% for both conditions was just over VT1 (71.1 ± 5.2%).

**Table 2.** Description of ventilatory thresholds and relative intensity of RE tests.

	**VT1**	**VT2**	**Free**	**Fix**
HR [b·min^−1^]	150.8 ± 11.45	173.9 ± 13.0	148.9 ± 11.9	148.8 ± 11.4
HR (% HRmax)	81.8 ± 5.7	94.1 ± 2.7	80.8 ± 6.4	80.7 ± 6.5
Threshold speed [km·h^−1^]	11.9 ± 0.9	14.8 ± 1.0	12	12
Threshold speed (%MAS)	71.5 ± 3.6	89.3 ± 3.3	72.7 ± 5.6	72.7 ± 5.6
Steady-state $\dot{V}$ O_2_ [mlO_2_·min^−1^·kg^−1^]	35.1 ± 3.5	43.6 ± 4.6	36.0 ± 2.1	36.1 ± 2.5
Steady-state $\dot{V}$ O_2_ (% of $\dot{V}$ O_2_ peak)	71.5 ± 3.6	89.3 ± 3.3	74.3 ± 0.1	74.3 ± 0.1
$\dot{V}$ E [l·min^−1^]	76.0 ± 8.9	108.0 ± 14.3	82.1 ± 12.4	81.1 ± 11.6

Free: measures without ROM limitations; Fix: measures with a ROM limited to (FE 90-45); HR: mean heart rate of the RE test; Steady-state $\dot{V}$ O_2_: oxygen consumption in the 2 last minutes of the RE test; RE: running economy; MAS: maximal aerobic speed; $\dot{V}$ E: minute ventilation.

### Effects of the ROM restriction

The restricted elbow ROM did not result in any alteration in participant exertion, as assessed by lactate levels, heart rate and oxygen consumption, showing no discernible difference. Regarding the main outcome, RE was not significantly different between the two conditions: 180 ± 10.6 mlO_2_·km^−1^·kg^−1^ versus 180.2 ± 12.3 mlO_2_·km^−1^·kg^−1^ with full ROM and with restricted ROM, respectively (*p*-value: 0.871; difference: 0.1%) (Table 3).

**Table 3.** Comparison of the performance depending on elbow movement.

	**Free**	**Fix**	***p*-value**
La (post) [mmol·^l−1^]	2.36 ± 0.88	2.24 ± 0.75	0.262
HR [b·min^−1^]	148.9 ± 11.9	148.8 ± 11.4	0.782
HR (% HRmax)	80.8 ± 6.4	80.7 ± 6.5	0.859
Steady-state $\dot{V}$ O_2_ [mlO_2_·min^−1^·kg^−1^]	36.0 ± 2.1	36.1 ± 2.5	0.871
Steady-state $\dot{V}$ O_2_ (% of $\dot{V}$ O_2_ peak)	74.3 ± 0.1	74.3 ± 0.1	0.999
RE [mlO_2_·km^−1^·kg^−1^]	180.1 ± 10.6	180.2 ± 12.3	0.871

Free: measures without ROM limitations; Fix: measures with a ROM limited to (FE 90-45); La(post): lactate level at the end of the 5 min-RE test; HR: mean heart rate of the RE test; steady-state $\dot{V}$ O_2_: oxygen consumption in the 2 last minutes of the RE test; RE: running economy; MAS: maximal aerobic speed.

## Discussion

In this study, we investigated the influence of severe elbow stiffness, characterized by restricted ROM, on the RE of experienced runners. We hypothesized that elbow stiffness would negatively affect RE in this population. However, our observational, crossover design involving submaximal running tests with both restricted and unrestricted elbow movements revealed no significant impact on RE, indicating that elbow stiffness may not compromise RE in trained recreational athletes. Moreover, post-traumatic elbow stiffness seldom induces such a severe loss of ROM.

Several studies have demonstrated that the global arm movement and its impact on vertical oscillation can affect the RE of athletes.^9,14,15^ Other studies have suggested that arm swing may not be crucial for running performance.^12,26^ Brooks et al. found that when all arm movement was restricted, the speed of a sprint runner was only moderately reduced (1.6%;  < 0.1 s).^26^ Pontzer et al. even showed in ten recreationally fit, healthy adults that running with no arm swing at 10.8 km·h^−1^ had no effect on the energetic cost of running.^12^ On the contrary, within a similar population and at equivalent speeds, Arellano and Kram demonstrated that running without arm swing elevated the net metabolic demand, consequently impeding the athlete's performance.^15^

Tartaruga et al. conducted the only study that specifically examined the elbow movement. In their study, the RE of a 16 highly trained male runners were compared and a kinetic analysis was performed.^6^ The results revealed a positive correlation between elbow ROM and RE, suggesting that runners with a smaller ROM exhibited better RE. While relevant, Tartaruga et al.'s study differs from ours in that they compared the performance of different individuals, whereas we compared the performances of the same individuals before and after our intervention, which involved modifying their ROM. When injured, a runner must adapt their biomechanics similarly to the participants in our study, making our research more relevant for understanding the impact of post-traumatic elbow stiffness on RE compared to the study conducted by Tartaruga et al.^6^

### Limitations

Only men were included in this study. Replicating this study in women may yield different results, as biomechanical and RE differences have been documented between sexes.^27^ Furthermore, the question of whether there is a difference if the limitation is imposed on the dominant hand or not can be raised. However, we have not found any studies that look at the impact of dominant hand on RE. Additionally, running on a motorized treadmill, as opposed to overground running, may induce biomechanical and physiological differences, despite being considered comparable overall.^28^

Although RE is a key physiological variable for endurance athletes, it is not the only one. In future research, other factors such as the influence on V˙O_2_max and the onset of VTs could be investigated.

Exploring the impact of elbow stiffness on different intensities of running would also be of interest, as sprint runners seem to have a bigger elbow ROM than long distance runners.^6,29^ Further studies could therefore assess the impact of elbow movement on shorter distance running. Moreover, to evaluate RE participants were running at a submaximal speed (12km·h^−1^), and the biomechanical effect of our intervention might be different at a higher speed. Finally, the study population exhibited excellent RE values. However, the impact of this intervention on less well-trained runners may differ.

In conclusion, our hypothesis that RE would be negatively impacted by a decreased ROM of the elbow was refuted in this sample under the conditions adopted for this study. No significant change in RE was observed when elbow mobility was limited at a speed of 12 km·h^−1^.

### Practical implications

Patients experiencing elbow stiffness and aiming to engage in moderate endurance running intensity may find reassurance in these findings, as this condition does not appear to exert a significant impact on one of the key indices of running performance.
